# Unveiling the complex relationship between gut microbiota and liver cancer: opportunities for novel therapeutic interventions

**DOI:** 10.1080/19490976.2023.2240031

**Published:** 2023-08-24

**Authors:** Jayashi Rajapakse, Saroj Khatiwada, Anna Camille Akon, Kin Lam Yu, Sj Shen, Amany Zekry

**Affiliations:** a UNSW Microbiome Research Centre, St George and Sutherland Clinical Campus, University of New South Wales (UNSW), Sydney, Australia; b St George Hospital, Gastroenterology and Hepatology Department, Sydney, Australia

**Keywords:** Gut microbiota, HCC, dysbiosis, gut-liver axis, antibiotics, Mediterranean diet, FMT, probiotics

## Abstract

Hepatocellular carcinoma (HCC) has been linked to the gut microbiota, with recent studies revealing the potential of gut-generated responses to influence several arms of the immune responses relevant to HCC formation. The pro- or anti-tumor effects of specific bacterial strains or gut microbiota-related metabolites, such as bile acids and short-chain fatty acids, have been highlighted in many human and animal studies. The critical role of the gut microbiota in HCC development has spurred interest in modulating the gut microbiota through dietary interventions, probiotics, and fecal microbiota transplantation as a potential strategy to improve liver cancer outcomes. Encouragingly, preclinical and clinical studies have demonstrated that modulation of the gut microbiota can ameliorate liver function, reduce inflammation, and inhibit liver tumor growth, underscoring the potential of this approach to improve HCC outcomes. As research continues to unravel the complex and dynamic mechanisms underlying the gut-liver axis, the development of safe and effective interventions to target this pathway for liver cancer prevention and treatment appears to be on the horizon, heralding a significant advance in our ongoing efforts to combat this devastating disease.

## Introduction

Hepatocellular carcinoma (HCC) is the most common type of primary liver cancer, representing 75–85% of the almost 906,000 liver cancer cases diagnosed in 2020 alone.^1^ HCC was also the third leading cause of cancer-related mortality worldwide in 2020, with an estimated 5-year survival rate of 20%.^2,3^

HCC is often a consequence of a range of chronic liver diseases which create a pro-tumorigenic environment through non-resolving inflammation and eventual suppression of the anti-tumor immune responses.^4,5^ These chronic liver diseases include alcoholic liver disease, nonalcoholic fatty liver disease (NAFLD) and chronic infection with Hepatitis B virus (HBV) and hepatitis C virus (HCV).^6^ Conditions such as obesity, insulin resistance and type II diabetes mellitus (T2DM), which often accompany NAFLD, have also been established as significant risk factors for HCC.^6^ Due to improved treatment options for HBV and HCV infections, NAFLD is emerging as a critical cause of HCC.^4^

The liver possesses an intimate bi-directional relationship with the gut microbiota known as the gut-liver axis. The microbiota and its metabolites can affect liver homeostasis, and disruption of normal gut flora balance, known as *dysbiosis*, can contribute to various liver pathologies.^6,7^ Dysbiosis of the gut microbiota has been linked to hepatic injury and inflammation through increased intestinal permeability and the subsequent translocation of bacteria and bacterial ligands.^7–10^

Several studies have demonstrated that patients with HCC have a different microbiome signature from non-HCC controls.^11,12^ In this setting, evidence supports the critical role of the gut microbiome and its metabolites in influencing several immune and metabolic events associated with HCC development and progression.^6^ This review will highlight the role of the gut microbiota in HCC, exploring its role in the pathogenesis of HCC and its potential in treating and preventing the disease.

## The gut microbiota in the pathogenesis of HCC

The molecular mechanisms linking the gut microbiota with liver cancer involve a complex interplay between microbial metabolites, host signaling pathways, and immune responses. Some key pathways through which the gut microbiome influences liver cancer are discussed below and summarized in Table 1. However, it is important to note that the interactions between the gut microbiome and liver cancer are complex and multifaceted, and further research is needed to fully understand the molecular intricacies of this relationship.

**Table 1.** Examples of molecular mechanisms linking the gut and HCC development and progression.

Molecules		Mechanism(s) of Action	References
Gut dysbiosis		Imbalance between commensal and pro-inflammatory bacteria and bacterial overgrowth → ↑ bacterial ligands and enterotoxins (e.g., LPS) → ↑ inflammation→ disruption of intestinal epithelial tight junctions and brush border → bacterial translocation into circulation and liver	^7–10,13–15^
Lipopolysaccharide and toll like receptor (TLR)		LPS/TLR4 signaling →activation of downstream NF-κB →↑ IL-6 and TNF-α → epithelial-to-mesenchymal transition → hepatocarcinogenesis	^8,15–20^
		↑ LPS → activation of TLR-4/MyD88 transduction pathway → ↑ MLCK expression → ↑ gut barrier permeability	
		Gut microbiota-derived ligands (e.g. LPS) activate TLR4 → ↑ epiregulin secretion by hepatic stellate cells, ↑ hepatocellular proliferation, inhibition of apoptosis → hepatocarcinogenesis	
		Leaky gut barrier allows LPS translocation→ ↑ intrahepatic pro-inflammatory/pro-tumor cytokines (e.g. TNF-α, IL-1β and CCR5) →↑ fibrosis + chronic inflammation + angiogenesis → promotes tumor survivability	
Bacterial metabolites	Short-chain fatty acids (SCFAs)	↓ anti-tumor immune response (↑ IL-10 production by Th1 cells, ↓ inflammatory macrophages)	^11,21–33^
		↑ pro-tumor hepatic inflammation and neutrophil influx → HCC development	
	Secondary bile acids (BAs)	Impaired BA signaling → suppressed activation of FXR → ↑ accumulation of fat in the liver and ↑ tumorigenesis	
		Impaired BA signaling → suppressed TGR5 activation → altered Treg activation	
		Induction of SASP in hepatic stellate cells → ↑ pro-inflammatory and pro-carcinogenic factors	
		Increased M2-like macrophage polarization → ↓ anti-tumor immune response	
		Impaired recruitment of anti-tumor immune cells (e.g., NKT cells) to tumor site	

### Gut integrity and microbiota composition in HCC

**Figure 1.**
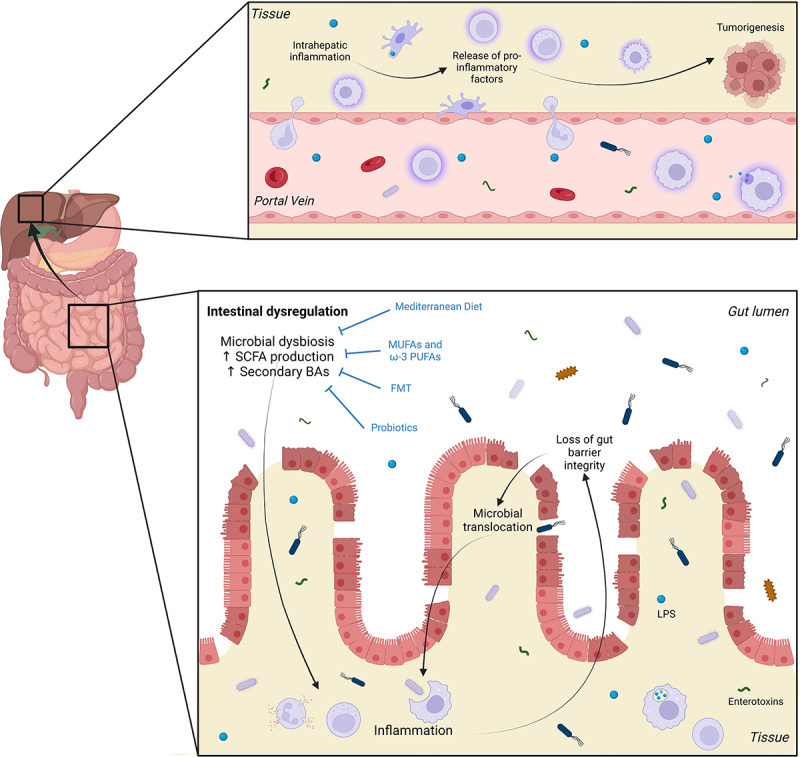
Events leading to initiation of inflammatory response in the gut and tumorigenesis in the liver. Intestinal dysregulation, characterized by disruption of normal gut flora balance and reduced microbial diversity (microbial dysbiosis), is associated with increased production of short chain fatty acids (SCFAs) and secondary bile acids (BAs). These factors can contribute to intestinal inflammation and the subsequent loss of gut barrier integrity through damage to epithelial tight junctions and the brush border. This, in turn, allows the translocation of bacteria and bacterial ligands such as lipopolysaccharide (LPS) and other enterotoxins, into the circulation. From here, these components can travel through the portal circulation to reach the liver where they catalyze chronic inflammation. The secretion of pro-inflammatory cytokines, chemokines, growth factors, prostaglandins and pro-angiogenic factors establishes an environment that is favorable to tumorigenesis. Interventions such as consumption of the Mediterranean diet and monounsaturated and omega-3 polyunsaturated fatty acids (MUFAs and PUFAs), as well as fecal microbiota transplantation (FMT) and probiotics have been linked to reduced HCC incidence due to their capacity to improve dysbiosis. Figure created with BioRender.com.

The specific mechanisms linking dysbiosis and inflammation are slowly unfolding. It is believed that an imbalance between commensal and pro-inflammatory bacteria during dysbiosis may lead to increased bacterial ligands and enterotoxins, triggering an inflammatory response in the gut.^7^ This may compromise gut barrier integrity by disrupting epithelial tight junctions (TJ) and the brush border.^7,9,10,13^ Increased intestinal permeability could be one mechanism that underpins direct effects of the gut microbiota on hepatic inflammation and pro-tumor responses (Figure 1). For example, elevated systemic levels of zonula occludens 1 (ZO-1), a TJ protein, correlated with increased intestinal permeability, inflammation, and disease severity in HCC patients.^10,14^ This increase in intestinal permeability may facilitate the translocation of microbial products like lipopolysaccharide (LPS) from the gut to the liver.^7,8^ LPS activates immune cells via toll-like receptor 4 (TLR4) and downstream nuclear factor kappa B (NF-κB) pathways, leading to pro-inflammatory cytokine production.^15^ During dysbiosis, bacterial overgrowth can stimulate pro-inflammatory cytokine production via the TLR4-NF-κB pathway, thus promoting intestinal inflammation and HCC progression.^16^ This is corroborated by studies demonstrating increased serum levels of LPS with progressive liver injury and HCC development.^8,10^ LPS may also act directly on intestinal epithelial cells to increase TJ permeability through TLR4/MyD88 signaling-mediated activation of myosin light chain kinase (MLCK), the overexpression of which has been shown to increase intestinal permeability.^17,18^

LPS promotes hepatic inflammation and HCC formation through several proposed mechanisms: Firstly, activation of LPS/TLR4 signaling has been shown to promote cell survival and proliferation in HCC by inducing the secretion of growth factors, such as epiregulin, by hepatic stellate cells (HSCs) which inhibited apoptosis and thereby promoted hepatocarcinogenesis.^15^ Accordingly, TLR4 inactivation, gut sterilization or germ-free status significantly decreased HCC development.^15^ In addition to HSCs, TLR4 activation affects various cell types, such as hepatocytes and Kupffer cells, leading to hepatocellular proliferation, inflammation, epithelial-to-mesenchymal transition, and angiogenesis in HCC.^15^ Secondly, in animal models of HCC, increased concentrations of LPS in the circulation are associated with increased intrahepatic pro-inflammatory mediators such as tumor necrosis factor (TNF)-α, interleukin (IL)-1β and C-C chemokine receptor (CCR)5, as well as increased tumor burden.^8,15^ Finally, LPS has recently been shown to induce malignant transformation of hepatic progenitor cells through increased production of IL-6 and TNF-α, a process that was mediated via LPS/TLR4 signaling.^19,20^

Due to the loss of gut barrier integrity, dysbiosis and subsequent alterations to the gut microbiome also contribute to systemic inflammation, which plays a role in liver cancer development.^5,34^ In patients with NAFLD-related HCC, the gut microbiota profile correlates with plasma levels of inflammatory mediators, including IL-8, IL-13, C-C chemokine ligand (CCL)3, CCL4, and CCL5.^12^ These findings highlight the significant correlation between the gut microbiota profile and systemic inflammation, which can synergistically contribute to the process of hepatocarcinogenesis.^12^

Concerning gut composition, several studies have demonstrated that the microbiota of patients with HCC differs from that of controls, with reduced alpha diversity and a greater degree of dysbiosis.^8,11,12,35^ In this setting, patients with HCC have an increased abundance of the *Parabacteroides*, *Clostridium* and *Gemmiger* genera and decreased prevalence of genera such as *Bifidobacterium* and *Lactobacillus* compared to patients without HCC.^8,12,36^ However, the specific compositional changes in the microbiota of HCC patients differ between studies, with conflicting reports on the abundance of many species.^37^ This may be due to differences in patient characteristics such as geographical location, ethnicity, nutrition, medication, chronic liver disease etiology and the extent of the underlying liver disease. A unique HCC-specific microbiota-based signature, consistent across studies regardless of ethnicity and underlying liver disease, is yet to be identified.

### Gut microbiota, its metabolites and the immune response in HCC

Gut microbiota-derived signaling compounds and metabolites can access the liver through portal circulation to influence the hepatic immune milieu, acting as an indirect mechanism by which the gut microbiota affects HCC. Liver sinusoidal endothelial cells (LSECs) are proposed as the primary interaction site with portal-delivered gut-derived pathogens.^38^ Upon sensing the gut microbiota, LSECs coordinate the localization of Kupffer cells and lymphocytes to provide efficient protection from infection.^39,40^ This gut-derived zonation of immune response is critical to limiting harmful inflammatory responses to the liver.^39,40^ Disruption of this critical gut-derived response contributes to liver inflammation.^39,40^ With chronic liver inflammation, the production of various cytokines, chemokines, growth factors, prostaglandins and pro-angiogenic factors creates a microenvironment supporting tumorigenesis.^5,34^ Further, as liver disease progresses and in the context of a dysbiotic environment, senescence surveillance of pre-malignant hepatocytes by T cells becomes impaired.^41^ Impaired immunosurveillance combined with an immunosuppressive milieu sets the liver microenvironment for cancer formation and progression.^8,11,42^

There is growing evidence for gut dysbiosis promoting an immunosuppressive milieu.^8^ In *ex vivo* studies, bacterial extracts from patients with HCC, when added to peripheral blood mononuclear cells (PBMCs) from healthy individuals, induced an immunosuppressed T cell phenotype characterized by expansion of regulatory T cells (Tregs) and attenuated development of cytotoxic CD8^+^ T cells.^11^ Moreover, in a mouse model of steatohepatitis-mediated HCC, animals with dysbiosis exhibited an intrahepatic immunosuppressive milieu characterized by elevated myeloid-derived suppressor cells (MDSCs) and a corresponding reduction in CD4^+^ and CD8^+^ T cells with a resultant increase in liver injury and HCC formation.^42^ This gut-mediated immunosuppressive response was further corroborated by human data confirming that the abundance of specific bacterial strains, such as the *Bacteroides* genus in HCC patients, correlated with increased MDSCs and the cytokines IL-8 and IL-13, which have roles in MDSC recruitment and proliferation, respectively.^12,43^ Additionally, gene expression analysis of livers from cirrhotic patients found that bacterial translocation into the liver, as measured by *16S* rRNA abundance in the liver, correlated with the expression of T cell exhaustion markers such as cytotoxic T lymphocyte – associated antigen (CTLA)4, programmed cell death protein (PD)-1 and thymocyte selection-associated HMG box (TOX), indicative of immunosuppression and impaired anti-cancer surveillance.^42^

The importance of the gut microbiota in mediating the anti-tumor immune response has been further shown through antibiotic treatment aimed at depleting gut commensal bacteria. In several animal studies, antibiotic-directed therapy at pathogenic bacteria was associated with reversing pro-tumor immunosuppressive events, restoring immunosurveillance, and consequently reducing liver tumor burden.^21,42^

The gut microbiota mediates the production of various metabolites which can directly or indirectly influence liver cancer development by modulating host signaling pathways and gene expression.^21,22,44,45^ Key gut metabolites associated with HCC include short-chain fatty acids (SCFAs) and bile acids (BAs).^7^

Concerning SCFAs, the most abundant SCFAs are acetate, propionate and butyrate, produced by the fermentation of undigested carbohydrates, amino acids, lactic acid and fibers.^46^ SCFAs can affect immune cell function, inflammation and cell proliferation through mechanisms such as histone deacetylase inhibition and activation of G protein-coupled receptors.^44^ Some studies reported decreased SCFA-producing bacteria in the feces of HCC patients, such as butyrate-producing *Lachnospira*, *Ruminococcus* and *Butyricicoccus* bacteria, suggesting beneficial roles of SCFAs in HCC.^47,48^ This is supported by murine studies of melanoma and pancreatic cancer, whereby pretreatment of CD8^+^ T cells and chimeric antigen receptor (CAR) T cells with the SCFA pentanoate, and to a lesser extent butyrate, led to decreased tumor volume and weight.^49^ This may have been mediated by increased tumor antigen-specific anti-cancer activity by these pretreated T cells, as evidenced by their enhanced proliferative capacity and higher expression of the pro-inflammatory cytokines, TNF-α and interferon (IFN)-γ.^49^

However, other studies have demonstrated increased SCFA concentrations in patients with HCC, suggesting a role in contributing to carcinogenesis.^11^ SCFAs mediate immunosuppression by promoting microbiota antigen-specific T helper (Th)1 cell IL-10 production or suppressing inflammatory macrophages in the lamina propria, causing reduced responsiveness to commensal bacteria.^23–25^ In dysbiosis, a high inulin diet increased the production of SCFAs, which was associated with liver inflammation, neutrophil influx and HCC formation in mice with elevated BAs and hyperbilirubinemia.^22,26^ The extent of liver injury in this context was ameliorated by antibiotics-mediated depletion of fiber-fermenting bacteria.^50,51^ Similarly, correlation analysis revealed that enriched SCFAs in the stools of subjects with NAFLD-related HCC was associated with an increase in peripheral blood Tregs and a decrease in cytotoxic CD8^+^ T cells.^11^ Collectively, these data support the notion that with dysbiosis, and advanced liver disease, increased SCFA production could be detrimental to liver disease as it promotes an immunosuppressive environment that could facilitate HCC progression. These recent findings highlight the intricate nature of the involvement of SCFAs in HCC development, indicating that their potential benefits are highly variable and likely influenced by various factors. These factors may include the stage of liver disease (early versus advanced), the degree of dysbiosis, and the presence of other metabolites, such as BAs. Therefore, further research is imperative to establish a causal relationship between SCFAs and immunosuppression in humans. Additionally, it is crucial to identify factors beyond dysbiosis and elevated BAs that contribute to SCFA-induced pro-tumor immunosuppression. Understanding the timing of these responses during the progression of liver disease and elucidating the underlying molecular mechanisms of this interaction are also important research objectives. Such investigations will not only advance our knowledge but will also facilitate the development of targeted interventions that modulate these metabolites, thereby preventing the development and progression of HCC.

Aberrant BA metabolism is being increasingly reported in HCC patients, predominantly due to alterations in the gut microbiota population.^52,53^ BAs are synthesized from cholesterol in the liver and are initially known as primary BAs, which include cholic and chenodeoxycholic acid (CA and CDCA, respectively).^54^ These BAs conjugate with taurine or glycine and are released into the duodenum.^54^ Once released, the gut microbiota act enzymatically on primary BAs to form secondary BAs, which include lithocholic acid and deoxycholic acid (LCA and DCA, respectively).^54^ Although most BAs act locally and are reabsorbed into the liver, a fraction remains in the systemic circulation and act as signaling molecules by activating nuclear receptors such as the farnesoid X receptor (FXR) and Takeda G protein-coupled bile acid receptor 1 (TGR5).^27,28^ Activation of FXR in the liver suppresses lipogenesis and enhances lipolysis, effectively preventing the accumulation of fat in hepatic cells. Notably, studies using liver-specific FXR-knockout mice have demonstrated a significant 20% incidence of HCC, highlighting the importance of FXR in HCC development.^29^ Additionally, the administration of obeticholic acid, a potent FXR agonist, has been shown to downregulate STAT3, which effectively limits the promotion of cancer.^30,55^ These findings indicate that the dysregulated accumulation of bile acids, coupled with suppressed FXR expression, may synergistically contribute to the process of carcinogenesis in the liver.^21^ In addition to FXR, another important membrane receptor involved in bile acid signaling is TGR5, which can be activated by specific bile acids such as ursodeoxycholic acid (UDCA).^31^ Activation of TGR5 has been implicated in the modulation of the anti-tumor immune response. In a murine model of tumor growth, the administration of a TGR5 agonist (INT777) or the administration of UDCA inhibited the activation of Tregs through the TGR5-AMPK-PKA (AMP-kinase, protein kinase A) axis.^31^ These findings underscore the involvement of TGR5 in regulating immune responses that may be relevant to the development of HCC.^31^

With varying activities between primary and secondary BAs, the balance between primary and secondary BAs is vital in controlling tumor growth and mediating anti-tumor response.^21,27,54^ In both mice and human studies, secondary BAs seem to promote an immunosuppressive environment. The gut microbiota’s conversion of primary BAs to secondary BAs regulated C-X-C motif ligand (CXCL)16 expression of LSECs and the migration of anti-tumor natural killer T (NKT) cells to the liver.^21^ To this effect, with increased secondary BAs LCA or ω-muricholic acid, LSECs expressed less CXCL16, with resultant impairment in the accumulation of NKT cells in the liver, whereas supplementation of CDCA resulted in an opposite response.^21^ Similarly, elevated secondary BAs also increased M2-like tumor-associated macrophage polarization, facilitating an immunosuppressive environment in the tumors.^32^ Additionally, in animal models of HCC, DCA caused HSC senescence, with induction of senescence-associated secretory phenotype factors (particularly IL-8 and transforming growth factor-β), creating a pro-inflammatory and carcinogenic environment.^33^ In another mouse model of HCC, antibiotic-mediated depletion of bacteria that favors the generation of secondary BAs was associated with reduced rates of HCC development.^50^ Collectively, these studies suggest that increased concentrations of secondary BAs can contribute to an immunosuppressive environment and have detrimental effects in the context of HCC.

Yet, emerging evidence suggest that this view may be oversimplified and not all secondary BAs are detrimental in HCC. For instance, the secondary BAs 3-oxo-lithocholic acid and isolithocholic acid were found to inhibit Th17 expression in mice.^56^ Such an immune profile is associated with better prognosis in HCC.^57^ Furthermore, it has been found that HCC responders to immunotherapy had a higher concentration of several fecal secondary BAs including UDCA, tauro-UDCA, ursocholic acid (UCA), and murideoxycholic acid (MDCA) as compared to non-responders.^58^ This evidence highlights the important role of BA signaling in linking the gut microbiota with immune responses relevant to HCC and treatment responses. However, more research is required to delineate the individual effects of each BA and their role in HCC pathogenesis.

Given the above data, it is unsurprising that research has shown that diets modulating the concentration of SCFAs and BAs can influence HCC formation. A notable example is the recently established link between diets high in fermentable fiber and HCC formation in animal models with cholestatic-induced liver injury. Two studies demonstrated that prolonged consumption of fermentable fiber, specifically inulin, predisposed mice to liver injury, hyperbilirubinemia, and HCC formation.^22,26^ The development of HCC in mice with a high concentration of BAs and hyperbilirubinemia was thought to be facilitated by the fermentation of these fibers by the gut microbiota into SCFAs. Increased SCFAs concentration in mice with high levels of BAs and hyperbilirubinemia led to reduced CD8^+^ T cells and increased Tregs and immunosuppressive immunoglobulin (Ig)A^+^ B cells in the liver, hence dampening the anti-tumor immune response.^26,44,59^ Similar results were also shown for other fermentable fibers, such as pectin and fructooligosaccharide, but not for non-fermentable fibers.^22^ In these studies, the detrimental effects of SCFAs on the development of HCC were mostly observed in the presence of elevated serum BAs and hyperbilirubinemia, the latter being induced by high fermentable fiber diets. Indeed, antibiotic-mediated depletion of SCFA and secondary BA-producing bacteria in these mice was correlated with the prevention of HCC tumor development.^22,50^ Thus, while SCFAs and BAs may have the potential to contribute to improved HCC outcomes, these benefits may be highly context-dependent, and an environment in which SCFA and BA production exceed levels tolerable by the host may ultimately prove detrimental due to the promotion of an immunosuppressive response that facilitates HCC formation and progression.

## Modulating the gut microbiome in hcc

Given the inextricable link between the gut microbiota and HCC pathogenesis, modulating the gut microbiota can affect HCC (Table 2). Hence, strategies to modulate the gut microenvironment present promising opportunities as noninvasive treatments for HCC and precursors such as NAFLD, as we previously reviewed.^7^ Some gut-modulating strategies are discussed below.

**Table 2.** Examples of gut-based interventions which can alter the course of HCC development and progression.

Intervention	Influence on HCC	Potential Mechanism(s) of Action	References
Antibiotics	↓ HCC growth in animals ↓ response to immunotherapy and ↑ mortality in humans	↓ commensal intestinal bacteria (e.g. Clostridium cluster XI and XIVa) → ↓ secondary BAs (e.g. DCA)→ ↓ senescent HSCs and ↑ hepatic NKT cell infiltrationDisruption of the gut-liver axis, ↑ dysbiosis Disruption of the gut-liver axis, ↑ dysbiosis	^21,50,53,60,61^
Mediterranean diet	↓ incidence of HCC and NAFLD	↑ diversity of gut microbiota → ↓ dysbiosis↓ circulating LPS ↓ circulating LPS	^62–72^
Monounsaturated fatty acids (MUFAs) and omega-3 polyunsaturated fatty acids (ω-3 PUFAs)	↓ HCC risk	↑ diversity of gut microbiota → ↓ dysbiosis↓ intestinal inflammationReversal of damage to intestinal mucosa ↓ intestinal inflammation Reversal of damage to intestinal mucosa	^73–83^
Fecal microbiota transplantation (FMT)	↓ hepatic inflammation and HCC risk ↑ effectiveness of immune checkpoint inhibitors → ↓ tumor growth	Altered microbiota composition → ↑ anti-tumor immune responses	^84–88^
Probiotics	↓ size and weight of liver tumors	↑ beneficial species, ↓ pathogenic species → ↓ intestinal inflammation↓ gut permeability → ↓ circulating LPS↑ binding of carcinogens ↓ gut permeability → ↓ circulating LPS ↑ binding of carcinogens	^89–94^

### Antibiotics

Antibiotic therapy in animal studies has demonstrated its capacity to modify gut microbiota composition and associated metabolites.^21,50,53^ Specifically, in obesity-induced HCC, the administration of an oral antibiotic cocktail effectively reduced commensal intestinal bacteria, resulting in a significant decrease in HCC development and the presence of senescent HSCs.^21,50,53^ Notably, the antibiotic vancomycin, which targets gram-positive bacteria, alone exhibited the ability to inhibit HCC development and the emergence of senescent HSCs.^53^ This outcome was achieved by depleting gut bacteria strains belonging to *Clostridium* cluster XI and XIVa, which are essential for increasing the concentration of DCA, a metabolite implicated in DCA-induced DNA damage – a precursor to the emergence of senescent HSCs.^53^

Likewise, in a cholestatic model of HCC, where mice were fed a high inulin diet, treatment with vancomycin effectively suppressed HCC development.^50^ This effect was attributed to the selective depletion of gut microbiota, including the reduction in secondary BAs responsible for creating a hepatocarcinogenic environment(as discussed above).^22^ Furthermore, in a mouse model of HCC with elevated expression of the *MYC* oncogene, the administration of an antibiotic cocktail consisting of vancomycin, neomycin, and primaxin yielded a notable reduction in both the number and size of HCC tumors compared to the control group.^21^ In this setting, the antibiotic treatment led to a significant expansion of hepatic C-X-X motif receptor (CXCR)6+ NKT cells in the liver, accompanied by elevated levels of IFN- γ, suggesting that depleting gut commensal bacteria enhances the anti-tumor function of hepatic NKT cells.^21^ Taken together, these findings highlight the significant impact of antibiotic-induced modifications to gut microbiota and associated metabolites on HCC development.

Contrary to the findings in animal studies, a growing body of evidence from human studies supports the notion that antibiotic treatment is an important outcome predictor in the context of immune checkpoint inhibitor (ICI) therapy.^60,61^ Recent studies have shed light on the impact of early antibiotic exposure on treatment outcomes in patients with HCC, not only in those treated with ICIs but also in those receiving tyrosine kinase inhibitors (TKIs), and placebo.

One study, analyzing data from nearly 4,100 patients with HCC participating in nine multicenter clinical trials, demonstrated that exposure to antibiotic treatment was associated with worse outcomes across all treatment groups.^60^ This finding suggests that the detrimental effects of antibiotics extend beyond the immunotherapy setting and may have broader implications in HCC management.^60^ In line with these results, a separate investigation conducted by researchers in Hong Kong evaluated data from 395 HCC patients who had received ICIs.^61^ The study revealed that concurrent antibiotic use during immunotherapy was linked to higher mortality rates in patients with advanced HCC.^61^ These findings reinforce the potential impact of antibiotics on treatment outcomes, specifically in the context of immunotherapy.

However, it is important to note that the causal relationship between antibiotic use and worse outcomes in HCC patients, potentially mediated through the disruption of the gut-liver axis, has yet to be definitively established. The potential disruption of the gut-liver axis as a mechanism underlying these observations warrants further investigation and could offer valuable insights into optimizing treatment strategies for HCC patients.

### Diets promoting anti-carcinogenic responses

Dietary modulation of the gut microbiome can favorably impact liver disease and HCC. One emerging dietary model in this area is the Mediterranean diet, which has been recognized for some time for its association with reduced risk of several conditions, including obesity, T2DM and NAFLD.^62,63^ The main characteristics of the Mediterranean diet are a beneficial fatty acid profile (specifically, low saturated fat and cholesterol, high monounsaturated and polyunsaturated fats) and increased consumption of complex carbohydrates, fibers and polyphenols.^62^ Various studies have established a link between adherence to a Mediterranean diet and improved metabolic variables associated with NAFLD, including the extent of hepatic steatosis, liver fat and insulin sensitivity.^62–66^ These outcomes were also independent of weight loss and associated factors such as energy intake and exercise, indicating that these improvements are linked to components inherent to the Mediterranean diet and not due to other health or lifestyle modifications. Through modulating well-known risk factors associated with NAFLD, this may prevent progression to cirrhosis and HCC.

Furthermore, population-based studies have shown that adherence to a Mediterranean diet among patients with liver cirrhosis was associated with a reduced incidence of HCC.^67^ Intervention with the Mediterranean diet also shifted toward a more beneficial and diverse gut microbiota composition, characterized by a reduction in bacterial species and metabolites associated with disease and a higher abundance of genera such as *Lactobacillus*, *Bifidobacterium* and *Faecalibacterium*.^68–70^ The increase in these genera is notable given that they are decreased in HCC.^68,69,95^ Other studies have also demonstrated that adherence to the Mediterranean diet is correlated with reduced circulating LPS levels, suggesting that this may be one mechanism by which the Mediterranean diet can reduce the risk of liver disease.^71,72^

It is important to note here that one characteristic of the Mediterranean diet is its high fiber content. As previously discussed, in the context of dysbiosis and the presence of high BAs, diets high in fermentable fiber can contribute to HCC pathogenesis.^22,26^ As such, in patients with dysbiosis, high BAs and hyperbilirubinemia, consumption of the Mediterranean diet may need to be modified so that levels of fermentable fiber are decreased.

Aside from the Mediterranean diet, a growing body of evidence suggests a relationship between the consumption of monounsaturated and omega-3 polyunsaturated fatty acids (MUFAs and ω-3 PUFAs, respectively) and HCC. Several observational studies have found an inverse correlation between the consumption of MUFAs and ω-3 PUFAs and the risk of HCC development.^73–76^ This protective effect may be mediated, at least in part, by the ability of MUFAs and ω-3 PUFAs to alter the composition of the gut microbiome, reduce intestinal inflammation and reverse damage to the intestinal mucosa.^77–81^ To this effect, mice fed diets rich in MUFAs and PUFAs have been shown to have higher microbial diversity and increased *Bifidobacteria*, often associated with protection against HCC.^77,82,83^

### Fecal microbiota transplantation

Fecal microbiota transplantation (FMT) has been proposed as a potential therapy for HCC, as it can modulate the gut microbiota and potentially reduce hepatic inflammation and the risk of HCC development.^84^ FMT effectively attenuated high-fat diet-induced steatohepatitis, as evidenced by a significant decrease in intrahepatic lipid accumulation and reduced expression of several intrahepatic pro-inflammatory cytokines, such as IFN-γ and IL-17.^85^ Further, in patients with alcoholic hepatitis ineligible for steroids, FMT from healthy donors alleviated manifestations of severe liver disease such as ascites and hepatic encephalopathy, as well as inflammatory markers.^96^

Another area where FMT may be beneficial is as an adjuvant therapy ICIs. As discussed above, patients who receive antibiotics alongside ICIs have been reported to have higher mortality, indicating that the gut microbiota has important roles in determining the effectiveness of ICIs.^60,61,97^ By shifting the gut microbiota composition, FMT has the potential to increase bacterial strains and metabolites associated with treatment response. In human studies of melanoma patients, FMT from responders to PD-1 blockade into non-responders resulted in improved response to PD-1 blockade.^86–88^ Responders demonstrated increased gut infiltration of antigen-presenting cells and tumor-infiltrating lymphocytes, suggesting a reinvigoration of the anti-tumor immune response.^87^ Although not studied in the context of HCC, FMT could enrich beneficial gut bacteria that promote ICI response. For example, the genus *Lachnoclostridium* is associated with enrichment of UDCA, tauro-UDCA, UCA, and MDCA, which are secondary BAs associated with response to ICI therapy in HCC.^58^ In addition, studies have linked the presence of the bacterium *Akkermansia muciniphila* to favorable treatment responses to ICI in several solid malignancies, including HCC.^98^ However, further research is needed to corroborate these findings and determine factors that shape the effectiveness of FMT in patients with HCC.

### Probiotics

Probiotics can be defined as “live microorganisms which, when administered in adequate amounts, confer a health benefit on the host”.^89^ Probiotics mainly achieve beneficial effects through alterations in intestinal immune reactions, interacting with microbes and generating metabolic end products (such as SCFAs).^89^ These mechanisms ultimately lead to the downregulation of potentially pathogenic microorganisms, strengthening of the mucosal barrier, anti-inflammatory effects and improved immune response to antigenic challenges.^89,90^ As such, it is now recognized that probiotics may be utilized to alter the trajectory of chronic liver disease complications, including HCC.

Multiple mechanisms explain the beneficial use of probiotics in HCC. It is proposed that probiotics can beneficially shift the gut microbiome to produce anti-inflammatory metabolites and downregulate receptors involved in liver inflammation and tumor angiogenesis.^89–91^

Feeding mice a novel probiotic mixture (comprising *Lactobacillus* species, *Escherichia coli* and heat-inactivated VSL#3) reduced Th17 cell infiltration into liver tumors with a resultant decrease in tumor weight and size and downregulation of angiogenic factors.^91^ These favorable changes were linked with the enrichment of specific genera of bacteria, including *Butyricimonas* and *Prevotella*, in probiotic-treated animals.^91^ Further, in a rat model of HCC, administration of VSL#3 resulted in decreased intestinal permeability and plasma LPS, correlating with reduced tumor weight and size.^92^

Evidence suggests that probiotics can also manipulate the binding and absorption of carcinogens. Aflatoxins are an established human hepatocarcinogen and a risk factor for hepatocellular carcinoma development.^99^ One study demonstrated that patients treated with a multi-strain probiotic (*Lactobacillus* and *Propionibacterium* species) had a lower level of urinary aflatoxins.^93^ Separate animal studies have established that these bacterial strains can bind aflatoxins and thus reduce their absorption.^94^ This reflects the utility of probiotics in reducing the harmful effects of dietary toxins and the risk of HCC.

The above evidence suggests that probiotics can be a cost-effective and noninvasive therapeutic against HCC. However, more clinical trials are needed to demonstrate the efficacy of probiotics in treating human HCC and to aid in selecting appropriate bacterial strains for use as probiotics. Further mechanistic studies must confirm how probiotics may influence HCC, especially in humans.

## Conclusion

The gut-liver axis refers to the intricate and dynamic interaction between the gut and the liver. Understanding the mechanisms by which gut-derived immune and metabolic signals influence liver injury and HCC formation remains an area of active research. Researchers hope to develop therapeutic strategies for liver disease and cancer by identifying these mechanisms and targeting the gut-liver axis. Such strategies may include antibiotics, probiotics, prebiotics, or dietary interventions to modulate the gut microbiota and improve liver function. Additionally, targeting immune and metabolic pathways that connect the gut and liver may offer promising avenues for treating liver disease and HCC.

In conclusion, the gut-liver axis is a complex and multifaceted system critical to liver health and disease. Recent research has highlighted the importance of this axis in the development and progression of HCC. Continued research in this area will be crucial for developing novel gut-based therapies that target the gut-liver axis and improve outcomes for patients with liver disease and cancer.
